# Certificated Dispensers and the Insurance Bill

**Published:** 1911-12-02

**Authors:** Montagu G. Smith

**Affiliations:** Chairman of Committee, Association of Certificated Dispensers. The Infirmary, 390 High Street, Lewisham, S.E.


*To the Editor of* The Hospital.

CERTIFICATED DISPENSERS AND THE INSURANCE BILL.

*To the Editor of* The Hospital.

Sir,—With reference to several letters that have appeared in The Hospital from persons who do not sign their names, I wish to make it clear that the Association of Certificated Dispensers has done all and a great deal more than your correspondents advise.

The persistent rushing into print at such a time does no good; in fact, a great deal of harm.

If these gentlemen are members of our Association they have not much faith in the Council which they elected. If they are not members, it behoves them to become so at once.

We do not require any assistance from the Press in the matter, for the Association is, and has been since the Insurance Bill was introduced, able to cope with every detail affecting its members' qualification. I ask all concerned to "wait and see," and take my word that the assistants' cause was never in better hands than at present, and that to cast the slightest slur upon the work of our esteemed Secretary, Mr. Albert Howell, is to acknowledge at least that they do not know him.

I am, Sir, yours faithfully,

Montagu G. Smith,

Chairman of Committee, Association of Certificated Dispensers.

The Infirmary,

390 High Street, Lewisham, S.E.,

November 27.

